# Comparative assessment of benzyl alcohol and m-cresol as preservatives in maropitant injectable formulation: implications for pain & tolerability

**DOI:** 10.3389/fvets.2026.1943767

**Published:** 2026-10-21

**Authors:** Vishwanath Pawar, Jinal Trivedi, Jayesh Muchhara, Nitin Dharvadiya, Satish Patel, Rajesh Patel, Miral Shah, Rajesh Sundar, M. E. Kannan, Mukesh Ukawala, Ritu Laddha, Mukul Jain

**Affiliations:** 1 Pharmacology & Toxicology, Zydus Lifesciences Limited, Ahmedabad, Gujarat, India; 2 Animal Research Facility, Zydus Lifesciences Limited, Ahmedabad, Gujarat, India; 3 Pharmaceutical Technology Centre (PTC), Zydus Lifesciences Limited, Ahmedabad, Gujarat, India

**Keywords:** beagle dogs, benzyl alcohol formulation, injection-site pain, maropitant, m-cresol, neurokinin 1 receptor

## Abstract

**Introduction:**

The selection of an optimal preservative in multi-dose injectable veterinary formulations requires antimicrobial efficacy with local tissue compatibility. While m-cresol provides potent broad-spectrum efficacy in multi-dose veterinary injectables, subcutaneous administration causes acute injection-site pain and distress, compromising compliance despite recommended low-temperature management. We hypothesized that a benzyl alcohol–preserved maropitant formulation (BAF) would result in lower acute injection-site pain scores than a *m-cresol* preserved formulation (MCF), regardless of storage temperature.

**Materials and methods:**

Thirty-two adult healthy beagle dogs were enrolled in this study. Under blinded, randomized crossover design, each dog received subcutaneous maropitant using both formulations maintained at refrigerated and room temperature conditions. Pain was assessed by two independent assessors using a visual analog scale, simple descriptive scale, and clinical observation. Cerenia^®^ was used as a meta-cresol-based formulation and benzyl-alcohol formulation was provided by ZyVet, USA.

**Results:**

The BAF produced significantly lower Visual Analog Scale (VAS) and Simple Descriptive Scale (SDS) scores than the MCF (*P* < 0.0001). Log VAS values for BAF were 1.94 ± 0.14 at 25 °C and 2.33 ± 0.14 at 4 °C, vs. 3.43 ± 0.11 at 25 °C and 3.38 ± 0.11 at 4 °C for MCF. Treatment with MCF increased the odds of elevated SDS scores up to 10.32-fold compared with BAF (*P* < 0.0001). Observer agreement was excellent. Restlessness and head shaking occurred more frequently with the m-cresol formulation.

**Discussion and conclusion:**

The benzyl alcohol–based maropitant formulation appears better-tolerated than meta-cresol containing formulation in terms of injection-site pain and irritation.

## 1 Introduction

In veterinary medicine, multi-dose injectable formulations require preservatives to maintain product integrity throughout repeated use (1). The selection of an appropriate preservative is an important factor influencing formulation stability and local tolerability following administration (1, 2). Commonly used preservatives include phenol, parabens, chlorobutanol, *m*-cresol, and benzyl alcohol (1, 2). Among these, *m*-cresol has been widely used because of its physicochemical stability and preservative effectiveness (1, 3, 4). However, its use has been associated with injection-site irritation and pain following subcutaneous administration (2, 5). Such local reactions may be particularly relevant for products requiring repeated administration (2).

To overcome these limitations, alternative preservative systems have been explored. Benzyl alcohol has emerged as a promising alternative for parenteral formulations because it possesses intrinsic local anesthetic properties that can mitigate injection-associated pain (2, 3). Maropitant, a selective neurokinin-1 receptor antagonist widely used as an injectable antiemetic in veterinary medicine, provides an appropriate clinical model for evaluating injection-site pain (5, 6). Although highly effective for treating emesis, commercially available maropitant formulations are well known to cause immediate pain and irritation sensations following subcutaneous injection (5), making them a sensitive benchmark for assessing factors that influence localized tolerability (2).

Despite these established individual observations, a significant knowledge gap persists in the existing literature regarding the relative, independent contributions of storage temperature vs. the structural composition of the preservative system on injection pain. In current veterinary practice, refrigeration is routinely recommended to mitigate the severe subcutaneous irritation associated with maropitant injections. However, it remains entirely unresolved whether this thermal intervention provides independent therapeutic relief, or if the localized pain is primarily driven by the choice of preservative chemical regardless of temperature. Previous studies have failed to decouple these variables, leaving a critical lack of comparative, controlled data regarding how these factors interact.

To address this knowledge gap and advance clinical understanding, this study evaluates a novel maropitant formulation containing benzyl alcohol as a preservative (BAF; ZyVet Animal Health Inc., Pennington, NJ, United States). This formulation was engineered to be qualitatively and quantitatively identical to the commercially available reference formulation (Cerenia^®^ Zoetis Inc., Parsippany, NJ, United States) in its primary physicochemical components, differing exclusively in the preservative system (11.8 mg/mL benzyl alcohol vs. 3.3 mg/mL meta-cresol, respectively). The scientific novelty of this study lies in its strict, isolated comparison of these two chemically identical vehicles across distinct thermal environments. Consequently, we hypothesized that the complete BAF formulation would demonstrate superior localized tissue tolerability and elicit lower immediate behavioral pain scores than the meta-cresol containing formulation (MCF). Furthermore, we hypothesized that the choice of the preservative system exerts the primary influence on immediate nociception, and we sought to systematically evaluate whether reducing the formulation temperature to 4 °C offers any additional clinical pain mitigation over room temperature (25 °C) administration for either product.

## 2 Material and methods

### 2.1 Animals

To check the pain/irritation response after the SC injection, 16 male and 16 female beagle dogs, more than 1 year of age and weighing between 8 and 12 kg, were used. Healthy dogs were selected based on based on a complete physical examination, [e.g., complete blood count (CBC), serum biochemistry profile]. Each dog was housed individually in individual floor kennel cages of approximately 2 square meter floor space. Each day, the floor of each kennel cage was cleaned to maintain hygiene. A controlled environment was maintained throughout the study, with a 12-h light/dark cycle, stable temperature, and regulated humidity. Standard laboratory animal diet (Pedigree^®^ Adult Small Breed; Mars Petcare, Mars International India Pvt. Ltd.) was provided once in a day during the housing. *Ad libitum* access to potable drinking water purified by a reverse osmosis system with subsequent UV treatment was provided. On the treatment day, food was given after completion of treatment and observation activities.

### 2.2 Test and reference item information

The reference product, Maropitant citrate injectable solution (Cerenia^®^, Zoetis, USA), was procured from the United States. Composition of Cerenia^®^: 10 mg/mL maropitant as maropitant citrate monohydrate, 63 mg/mL sulphobutylether-β-cyclodextrin, and 3.3 mg/mL meta-cresol, pH: 4.4, Osmolality: 290–310 mOsmol/Kg (7). The ZyVet product was synthesized in-house and which has composition as follow: 10 mg/mL maropitant as maropitant citrate monohydrate, 63 mg/mL sulphobutylether-β-cyclodextrin, and 11.8 mg/mL benzyl Alcohol, pH: 4.1 to 4.7, Osmolality: 250–350 mOsmol/Kg. this data is reported as a result of analysis of Zyvet formulations.

### 2.3 Experimental procedure

The pain assessment experiment was conducted using a double-blinded, randomized, crossover design structured as a four-period, four-sequence, four-treatment Latin square (Table 1). All procedures adhered to Good Laboratory Practice (GLP) standards. Randomization was carried out separately for male and female dogs using a random number generator in Microsoft Excel and divided into four groups (each of having 8 dogs: 4 male & 4 female). Each subject animal received four subcutaneous administrations of maropitant at 1 mg/kg, with a minimum washout of 3 days between treatments. Single dose of Test article or Reference article was administered at approximately the same time on the respective treatment days. Both the formulations were administered subcutaneously using a 26_1/2_ G needle at designated sites on the clipped dorsolateral flank, approximately 2.5 cm lateral to the spine. Injection sites were rotated systematically across study periods: cranial right in period I, caudal right in period II, caudal left in period III, and cranial left in period IV.

**Table 1:** Latin square crossover design matrix.

Sequence	Group (*N* = 8)	Period I	Period II	Period III	Period IV
A	1	BAF (4 °C)	MCF (25 °C)	MCF (4 °C)	BAF (25 °C)
B	2	BAF (25 °C)	BAF (4 °C)	MCF (25 °C)	MCF (4 °C)
C	3	MCF (4 °C)	BAF (25 °C)	BAF (4 °C)	MCF (25 °C)
D	4	MCF (25 °C)	MCF (4 °C)	BAF (25 °C)	BAF (4 °C)

To achieve the required temperature conditions, vials of each formulation was equilibrated for 1.5–2 h prior to dosing. One set was maintained in an ice bath at 4 ± 2 °C, while the other was placed in a water bath at 25 ± 2 °C. The temperature of the equilibration baths was continuously monitored using a calibrated digital thermometer, and injections were administered within 30 s of withdrawal from the vial. A dedicated needle was used to withdraw the product from the temperature conditioned vials, and a fresh sterile needle was employed for each injection to ensure consistency and minimize variability. The dose volume for test and reference article was 0.1 mL/kg. Volume of dose administered was calculated based on body weight recorded within 7 days of treatment.

### 2.4 Pain assessment

Following dosing, the administering personnel, who was blinded to treatment allocation, exited the enclosure, after which pain assessment was immediately conducted for 2 min by blinded observers using three pain assessment scales: Visual Analog Scale (VAS), Simple Descriptive Scale (SDS), and clinical observation. Injection pain (VAS and SDS) was assessed independently by two observers (for each observation), blinded to treatment allocation. Prior to study initiation, evaluators received standardized training in pain assessment methodology, with emphasis on identifying canine nociceptive responses using predefined clinical parameters and behavioral indicators. This calibration process was implemented to enhance inter-observer reliability and minimize variability in scoring.

Immediately after injection, pain was assessed for 2 min by two observers using three parameters: the Visual Analog Scale (VAS), the Simple Descriptive Scale (SDS), and clinical observation. VAS involved marking pain intensity on a 10-cm line (0 = no pain, 10 = maximal pain) (8, 9), while SDS categorized behavioral responses from 0 (no reaction) to 3 (severe response) as per Table 2 (2, 10). Clinical indicators such as restlessness, head shaking, or local reactions were also recorded (5, 11). At 24 h post-injection, sites were examined for delayed reactions including swelling or tenderness.

**Table 2:** Description of SDS score.

SDS score	Description
0	No reaction to injection
1	Mild reaction (e.g., twitching of the skin, looking at injection site, one-time licking, or scratching of the injection site)
2	Moderate reaction (e.g., repeated licking or scratching, short term vocalization, jumping, or shuddering)
3	Severe reaction (e.g., prolonged yelping, hiding, or circling with tucked tail or aggression)

All statistical evaluations were conducted using SAS^®^ software (version 9.4). Visual analog scale (VAS) scores were analyzed through analysis of variance (ANOVA) (PROC GLM) employing a fixed effects in the model: sequence, subject within sequence, period, and treatment. To meet model assumptions, the mean VAS scores from the two observers were subjected to logarithmic transformation prior to analysis. Treatment means were estimated using least square means derived from the model. Simple descriptive scale (SDS) scores were assessed using an ordinal logistic regression approach (PROC GENMOD), with fixed effect model as carried out for VAS. When treatment effects reached statistical significance within these models, pairwise comparisons were subsequently performed. Two-sided testing was applied across all pairwise treatment evaluations, and statistical inferences were drawn using the individual dog as the fundamental experimental unit. Inter observer reliability for SDS scoring was quantified using Cohen's kappa statistic, while agreement between observers on VAS scoring was evaluated using Lin's concordance correlation coefficient (ρc). The strength of agreement for kappa was interpreted as fair (0.21–0.40), moderate (0.41–0.60), substantial (0.61–0.80), and almost perfect (0.81–1.00). For Lin's concordance correlation coefficient, agreement was classified as fair (>0.70–0.80), moderate (>0.80–0.90), substantial (>0.90–0.95), and excellent (>0.95–1.00).

### 2.5 Animal ethics statement

All animal procedures in this study were conducted in accordance with institutional and national guidelines for the care and use of laboratory animals. Outside experimental days, dogs were socialized for two hours in a play area to encourage enrichment and natural behaviors. The experimental protocol (IAEC Protocol No. ZRC/DMPK/NP/185/07-2K25) was approved by the Institutional Animal Ethics Committee (IAEC) of Zydus Research Centre, followed by Committee for the Control and Supervision of Experiments on Animals (CCSEA), Government of India.

## 3 Results

Sample size determination was based on historical maropitant injection-pain studies. Assuming a clinically relevant difference in VAS scores between formulations, a power of 80%, and a two-sided α-level of 0.05, 32 dogs were considered sufficient to detect treatment-related differences. Pairwise treatment comparisons using Tukey-adjusted least-square mean differences of log-transformed VAS scores demonstrated that administration of BAF at ambient temperature (1.94 ± 0.14 at 25 °C) as well as under refrigerated conditions (2.33 ± 0.14 at 4 °C) resulted in values that were statistically lower than those observed following administration of MCF at either 25 °C (3.43 ± 0.11) or 4 °C (3.38 ± 0.11) (Table 3). Within each formulation comparisons, revealed no significant differences between the two temperature conditions for BAF or for MCF, indicating that the thermal state of the formulations did not materially influence pain perception as measured by VAS. Concordance between the two independent observers was exceptionally high, with Lin's concordance correlation coefficient (ρc) calculated at 0.99, thereby confirming excellent inter-observer agreement. The distribution of mean VAS scores across treatment groups is presented graphically in Figure 1.

**Table 3:** Log transformed LS means of Visual analog scale scores comparing injection pain after subcutaneous injection of two formulations of maropitant solution for injection (at 4 °C and 25 °C) to 32 beagle dogs.

Treatment (*n* = 32)	BAF (4 °C)	MCF (4 °C)	BAF (25 °C)	MCF (25 °C)
Least square Mean ± Standard error	2.33 ± 0.14^*^	3.38 ± 0.11	1.94 ± 0.14^*^	3.43 ± 0.11

^*^p < 0.0001, BAF (4 °C) vs. MCF (4 °C), BAF (25 °C) vs. MCF (25 °C), ANOVA (PROC GLM) followed by Tukey's multiple comparison post-hoc test.

**Figure 1:**
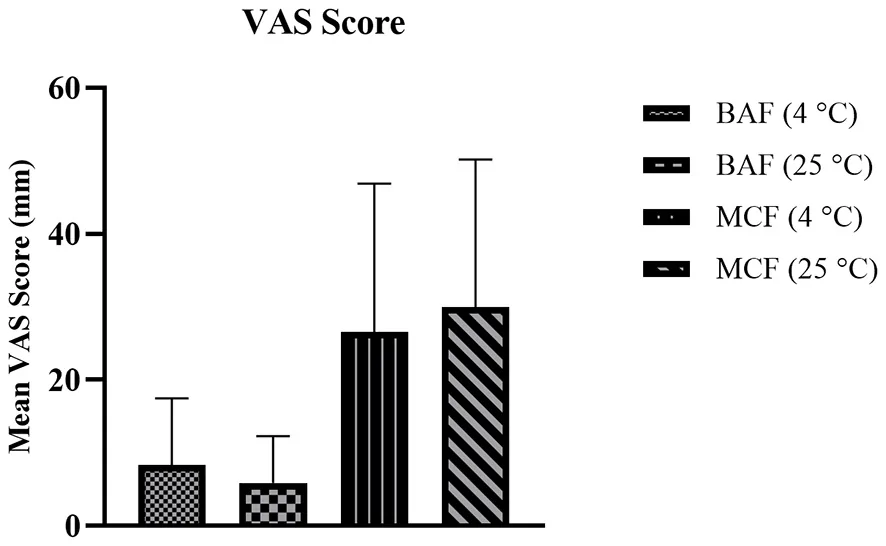
Mean visual analog scale scores (in mm) comparing injection pain after subcutaneous injection of two formulations of maropitant solution for injection (at 4 °C and 25 °C) to 32 beagle dogs.

Evaluation of SDS scores further substantiated these findings, with dogs receiving BAF at both 25 °C and 4 °C exhibiting significantly lower ordinal pain scores compared with those administered with MCF under either condition. Specifically, treatment with MCF at 25 °C was associated with a 10.32-fold increase (95% CI: 3.75–28.4) in the odds of higher pain scores relative to BAF at 25 °C, and a 6.51-fold increase (95% CI: 2.42–17.56) relative to BAF at 4 °C. Similarly, administration of MCF at 4 °C yielded odds ratios of 7.61 (95% CI: 2.84–20.38) and 4.80 (95% CI: 1.83–12.62) when compared with BAF at 25 °C and 4 °C, respectively. Although a slight elevation in pain scores was noted for BAF at 4 °C compared with 25 °C [odds ratio 1.58 (95% CI: 0.60–4.18)], this difference did not reach statistical significance (*P* = 0.35). Inter-observer reliability for SDS scoring was classified as almost perfect, with Cohen's kappa coefficient (κ) calculated at 0.89. Summary statistics for SDS scores, including mean, median, and range, are provided in Table 4.

**Table 4:** Simple descriptive scale scores (mean, median, and range) for 32 beagle dogs receiving subcutaneous injections of two formulations of maropitant solution for injection at 4 °C and 25 °C.

Treatment (*n* = 32)	Mean	Median (range)
BAF (4 °C)	0.8	1 (0–2)
BAF (25 °C)	0.6	0 (0–2)
MCF (4 °C)	1.4	1 (0–3)
MCF (25 °C)	1.5	2 (0–2)

Descriptive evaluation of clinical signs, although not subjected to formal statistical testing, revealed a clear disparity in frequency between formulations, with a total of 68 events recorded following m-cresol preserved formulation administration compared with 18 events after BAF. The most prevalent reactions consisted of mild restlessness and head shaking. Out of total 32 dogs' restlessness was observed in 25 dogs treated with MCF (11 at 4 °C and 14 at 25 °C) compared with 6 dogs treated with BAF (3 at each temperature). Head shaking was documented in 15 dogs following MCF (8 at 4 °C and 7 at 25 °C), whereas only a single occurrence was noted in the BAF 4 °C group. A detailed summary of clinical signs observed during and immediately after injection is presented in Table 5.

**Table 5:** Frequency of clinical signs observed during and within 2 min after subcutaneous injection of two formulations of maropitant solution to 32 beagle dogs at 4 °C and 25 °C.

Clinical Signs	BAF (4 °C)	BAF (25 °C)	MCF (4 °C)	MCF (25 °C)
Hunched or cramped posture	0	0	0	0
Shaking head	1	0	8	7
Scratching injection site	2	1	2	2
Stereotype behavior	1	0	2	3
Slight aggressive behavior	1	1	1	4
Slight tremors/muscle twitching	0	0	0	1
Quick breathing	1	0	1	0
Slight restless behavior	3	3	11	14
Lateral recumbency	0	0	0	0
Howling	0	1	0	0
Squeaking/Squealing	1	2	3	9
Total	10	8	28	40

At the 24-h post-injection evaluation, no evidence of local adverse reactions, including swelling, erythema, or pain upon palpation, was detected at the injection-sites in any of the treatment groups, thereby indicating an absence of delayed inflammatory or nociceptive responses.

To confirm the methodological validity of the 4 × 4 crossover model, potential confounding design effects were evaluated for both clinical endpoints prior to analyzing formulation performance. For the continuous endpoint, Analysis of Variance (ANOVA) via PROC GLM, applied to log-transformed mean VAS scores from two observers, demonstrated that the trial layout remained unaffected by operational biases. The structural variance linked to sequence assignment [*F*_(3, 92)_ = 0.28, *P* = 0.840], period progression [*F*_(3, 92)_ = 0.81, *P* = 0.492], and subjects within sequences [*F*_(28, 92)_ = 1.11, *P* = 0.345] were all highly non-significant. This absence of methodological confounding was uniformly mirrored in the categorical severity trends analyzed via ordinal logistic regression (PROC GENMOD) for the Simple Descriptive Scale (SDS). The cumulative logit models confirmed that the likelihood of scoring in higher severity brackets was entirely independent of Sequence [Wald χ^2^(3) = 1.05, *P* = 0.789], Period [Wald χ^2^(3) = 2.94, *P* = 0.401], and Subject within Sequence [Wald χ^2^(28) = 24.12, *P* = 0.675]. Collectively, these concurrent findings across both tracking metrics verify that no statistically significant sequence, period, or subject-within-sequence effects were detected, suggesting that these factors did not materially influence treatment comparisons.

## 4 Discussion

The selection of an optimal preservative system in multi-dose veterinary injectables requires balancing robust antimicrobial efficacy and localized tissue tolerability (12). While m-cresol has long been favored in veterinary formulations for its potent antimicrobial property and physicochemical stability, its clinical utility is frequently undermined by immediate, acute injection-site pain and localized distress in companion animals (2, 12). This study utilized maropitant—a highly effective neurokinin-1 (NK1) receptor antagonist associated with severe subcutaneous stinging—as a sensitive benchmark molecule to evaluate the local tolerability of a BAF (a ZyVet product) against the market meta cresol containing formulation (Cerenia^®^) under different thermal conditions (2). Both formulations shared an identical composition except for the choice of preservative.

Our findings demonstrate a clear, statistically significant divergence in injection-site pain scores between the two formulations (2). Dogs receiving the BAF consistently exhibited lower pain scores, as assessed by both the VAS and SDS, compared to those administered with MCF. Rather than relying solely on abstract numeric metrics, these lower scale scores corresponded directly to a marked reduction in observable clinical signs and acute procedural distress. On the SDS, MCF administrations frequently triggered overt clinical manifestations of pain, including scratching of the injection site, short term vocalization and prolonged post-injection site-directed attention (licking or scratching at the injection site). Conversely, BAF injections typically resulted in minimal to no vocalization and passive compliance during administration. On the continuous line of the VAS, independent observers recorded narrower, lower-bound distributions for BAF, which correlated clinically with a lack of systemic stress indicators. Furthermore, the acute local tissue tolerance mirrored these behavioral findings; dogs in the MCF cohort exhibited higher frequencies of restless behavior, shaking head, and squeaking/squealing within the first 2 min post-injection, signs that were largely absent or drastically diminished in the BAF cohort. This outcome strongly supports the hypothesis that the inclusion of benzyl alcohol mitigates the acute localized distress typically observed following currently available maropitant formulation (2, 13).

The pain-mitigating effect of benzyl alcohol observed in our study aligns with historical data across both human and veterinary medicine, where its secondary role as a local anesthetic has been utilized to improve formulation tolerability (13, 14). In human therapeutics, the addition of benzyl alcohol to injectables—such as clindamycin phosphate, certain localized vaccines, and even bacteriostatic water for injection—has been documented to markedly decrease patient-reported pain scores compared to preservative-free or alternative-preserved cohorts (14, 15). In veterinary clinical applications, similar analgesic trends have been reported; for instance, the incorporation of benzyl alcohol in certain multi-dose bovine and canine antibiotic formulations (such as specific penicillin or macrolide preparations) historically resulted in fewer physical avoidance behaviors, less vocalization, and diminished localized inflammatory swelling upon subcutaneous or intramuscular injection (5, 13).

The observed divergence in pain scores may be linked to the distinct pharmacodynamic and physicochemical properties of the two preservatives. Literature indicates that benzyl alcohol may contribute to local anesthetic effects due to its ability to transiently block voltage-gated sodium channels and stabilize neuronal membranes along nociceptive afferent fibers (14, 16). This transient numbing effect could theoretically suppress the peripheral nociceptor excitability and substance P pathways triggered by maropitant injection (17, 18). Furthermore, their structural characteristics may influence tissue tolerability. Benzyl alcohol possesses a moderate cLogP (~1.1) and high pKa (~15.4), remaining structurally neutral at physiological pH (12, 19). Conversely, meta-cresol features a higher lipophilicity (cLogP ~2.0) and a more reactive, acidic phenolic structure (pKa ~10.1), which may facilitate rapid cellular membrane partitioning, potentially amplifying baseline tissue irritation and subsequent nociceptor activation without any compensatory anesthetic capacity (5, 12, 19).

It has been widely hypothesized in veterinary clinical practice that refrigerating Cerenia^®^ stabilizes its temperature-sensitive cyclodextrin-maropitant inclusion complex, thereby minimizing the fraction of free, unbound drug in solution, and reducing local sting (15). However, our findings have clearly revealed that no statistically significant effect of storage temperature was observed within either formulation under the conditions of this study (2). Both formulations demonstrated comparable pain responses when administered at refrigerated (4 °C) and room temperature (25 °C). Importantly, the superior tolerability profile of the BAF remained evident even when administered at 25 °C, whereas MCF remained significantly more painful despite being cooled to 4 °C. This critical finding indicates that the specific chemical composition of the preservative exerts a far more powerful influence on immediate nociception than the physical thermal state or the theoretical stability of the cyclodextrin complex alone (2, 5). Consequently, while cooling a formulation may alter its physical delivery dynamics, it cannot overcome the inherent tissue irritation caused by an excipient like m-cresol, nor can it match the active, pain-mitigating anesthetic properties provided by benzyl alcohol (2).

In a previously published study evaluating pain scores for maropitant injectables, a significant period effect was reported, which the authors attributed to shifting injection-site locations and variations in animal restraint techniques across sequential treatment blocks (2). Specifically, more caudal scruff administrations between the scapulae correlated with elevated pain scores and localized swelling (2). In contrast, our study revealed no evidence of a period effect, nor did it show any significant sequence or subject-within-sequence effects. Across all treatment periods, pain scores remained uniform and stable. The absence of these statistical variations highlights the strict standardization protocols enforced throughout our trial design, which included uniform injection-site selection and consistent, trained personnel managing physical restraint. By eliminating these operational, order-dependent, and subject-specific confounders, we minimized experimental noise and confirmed that the observed differences in pain metrics were driven strictly by the intrinsic properties of the formulations.

While this study was conducted under strict GLP-compliant conditions, several limitations and potential confounding factors should be acknowledged. First, although assessment bias was minimized by utilizing a double-blind design and two independent observers, the Visual Analog Scale (VAS) and Simple Descriptive Scale (SDS) remain inherently subjective, meaning subtle variations in individual dog temperament or localized pain tolerances could introduce minor variability into the acute behavioral scores. Second, our study population was restricted to healthy, purpose-bred laboratory Beagle dogs; while this uniform cohort drastically reduced background environmental variables, laboratory Beagles are frequently handled and may exhibit more stoic behavioral phenotypes than a diverse, clinical population of client-owned companion animals. Third, local tissue site evaluation was concluded at 24 h post-injection, which captured acute local reactions like immediate swelling or pain on palpation but did not monitor for potential long-term, delayed focal tissue reactions or subclinical histological changes that could further differentiate the safety profiles of the meta-cresol and benzyl alcohol preservatives.

In conclusion, the benzyl alcohol-preserved formulation (BAF) significantly minimizes acute injection pain and localized irritation in healthy beagle dogs compared to m-cresol formulations. This distinct clinical advantage in patient comfort is maintained independently of storage temperature, while fully satisfying regulatory antimicrobial mandates for multi-dose vials. Further studies in real world patient data in clinical settings would be useful to substantiate these findings.

## Data Availability

The original contributions presented in the study are included in the article/supplementary material, further inquiries can be directed to the corresponding author.
